# An automated approach to extracting head and brain circumference from MRI datasets

**DOI:** 10.1371/journal.pone.0352445

**Published:** 2026-07-27

**Authors:** Jasmin Klischat, Marko Wilke

**Affiliations:** 1 Department of Neuropediatrics, General Pediatrics, Diabetology, Endocrinology, Social Paediatrics, University Children's Hospital, Tübingen, Germany; 2 Experimental Pediatric Neuroimaging, Children’s Hospital and Department of Neuroradiology, University of Tübingen, Germany; Massachusetts General Hospital, UNITED STATES OF AMERICA

## Abstract

Head circumference is a fundamental biometric parameter for brain growth in both the clinical pediatric setting and in developmental neuroscience. However, the gold standard for obtaining head circumference by manual tape measurement is notoriously error-prone. Further, while it is known that the growth trajectories of head and brain differ over time, a systematic comparison of these two parameters as a function of age does not yet exist. We developed a new and automated algorithm for obtaining head and brain circumference from MRI data. The algorithm mimics manual head circumference measurement by placing a convex hull around axial slices which must intersect with predefined anatomical landmarks. Several differently-tilted iterations are run and results are combined. In addition to obtaining head circumference, the approach can also be applied to gray matter only, providing “brain circumference” (gray matter hull perimeter) at the same level as head circumference. To assess validity, we used T1-weighted 3D datasets (n = 153) with available, manually measured head circumference values (age range 0–226 months [0–18.8 years]). To assess test-retest reliability, a second dataset (n = 3 with 40 scans each) was used. When compared with the current gold standard (manual measure), high validity was demonstrated for the new approach, with no systematic bias. The algorithm also showed a very high reliability across multiple measurements. Developmental trajectories of both head and brain circumference were generated and compared. In summary, the algorithm represents a valid and reliable method for the automated determination of head as well as brain circumference. It offers an objective way to assess these parameters in retrospect and prospectively, and may shed light on specific clinical situations where they differ, such as in the presence of enlarged subarachnoid spaces.

## Introduction

The human skull undergoes great structural and geometric changes in the first two years of life [1], reflecting the considerable changes in its contents. For example, there is a doubling of intracranial volume within the first six to nine months of life and a further 20% increase in size follows in the subsequent six months [2]. These substantial changes reflect the fact that a large part of human brain maturation is postnatal [3].

Consequently, head circumference is considered a fundamental biometric parameter for brain growth in both the clinical pediatric setting and in developmental neuroscience [4]. Head circumference measurements are also of multidisciplinary interest: for pediatricians, developmental neurologists, in therapy decisions for oral and maxillofacial, plastic or neurosurgeons, and also for anthropological studies [1].

When the term “head circumference” is used, it usually refers to the maximum occipitofrontal circumference which can be measured in different ways. Prenatally, sonography can be used, applying an ellipse function in the transthalamic cross-sectional plane [5,6]. Alternatively, the head circumference can be determined from two orthogonal head diameters [7]: The biparietal diameter (BPD) represents the maximum cranial width, the occipitofrontal diameter (OFD) the maximum longitudinal extension (i.e., cranial length). Postnatally, the most widely used method is by manual tape measurement, determined by placing the tape measure supraorbitally at the forehead level, and occipitally on the occipital protuberance [5]. Three-dimensional optical measurement options are a comparatively new method for (postnatal) determination of the head circumference, including laser scanners or 3D camera systems. However, the precision of the individual measurement of mobile scanners is not yet adequate for clinical use [8].

Manual tape measurement therefore is considered the gold standard and has the advantage of obtaining head circumference in a simple, quick, and inexpensive way, thus allowing to estimate brain volume in developing children [9,10]. However, several aspects of the measuring tape approach are problematic. First, there are no universally accepted standardized guidelines for measuring head circumference [4]. For example, while the WHO recommends placing the measuring tape directly above the eyebrows in its measurement protocol [11], other authors [12] points out that this position does not always reflect the largest occipitofrontal extension of the head. Also, the measurement is complicated by individual differences in head shape, volume of hair, and cooperation of the person being examined, as well as by the examiner with regard to the measurement location and the tightness of the measuring tape applied [4]. Due to these uncertainties, it is commonly recommended to measure several times in succession [5,10] in order to obtain a more representative result. Thereafter, though, there is again no consensus with regard to using the mean value, such as in [4,13] or in [14], or the maximum value, such as in [15,16, 17]. Finally, the exact practical procedure for measuring head circumference is often not reported [9,18], and it was observed that in many cases of supposedly “too small circumference”, measurement errors are to blame [16].

In addition, there are various norm value curves that are used when evaluating the measured head circumference [5], and norm values from one population [19] may not be suitable for children from, e.g., industrialized countries [12,20]. Additionally, it was pointed out [21] that a two-dimensional head circumference measurement has its shortcomings when assessing three-dimensional deformities, such as those that occur more frequently in premature infants. Consequently, infants with the same head circumference can still have different total brain volumes.

Macrocephaly is a good example of the clinical application and significance of head circumference. It is a relatively common clinical presentation and affects up to 5% of children [22], defined as a head circumference greater than two standard deviations (SD) above the mean, or greater than the age- and sex-specific 97th percentile [23]. The causes of macrocephaly are very heterogeneous. While there is a close correlation between microcephaly and micrencephaly (a particularly small brain), megalencephaly is only one possible cause of macrocephaly [24]. Macrocephaly is therefore not necessarily associated with an enlarged brain. In fact, it usually is not, as a common cause of macrocephaly [24] are disorders of cerebrospinal fluid (CSF) circulation, such as benign enlargement of subarachnoid space in infancy (BESS) and, of course, internal hydrocephalus [25,26]. BESS is the most common cause of macrocephaly in childhood with an estimated incidence of 0.4 per 1000 live births [27]. The majority of children show no clinical signs or symptoms of increased intracranial pressure and are physically, neurologically and developmentally unremarkable at follow-up [24]. Overall, the condition is considered self-limiting, with a rapid increase in head circumference around the age of 6 months, stabilization around the age of 18 months and a spontaneous (relative) decline around the age of 3 years [28]. Only few of these children show temporary developmental delays in language, gross motor, social or cognitive skills [29]; hence, surgical or medical therapy is rarely used and is not usually recommended [25,27]. It is, however, often difficult to differentiate benign from not-so-benign hydrocephalus early in the course of clinical presentation. In this setting, reference values for not only head circumference (which may be too high), but also brain circumference (which may be normal) would be helpful.

The aim of this study was to develop and evaluate an automated algorithm for determining head and brain circumference using MRI data from healthy subjects. With regard to validity, the impact of using the mean, median, or maximum value from several measurements was assessed. With regard to reliability, the reproducibility of results across several measurements was assessed. As the algorithm provides not only head but also brain circumference, the developmental trajectories of both parameters (and their relation) also were investigated.

## Subjects and methods

To assess validity, data from the C-MIND study (Cincinnati MR Imaging of Neurodevelopment study) [30] was used. This validation dataset (n = 153) with available, manually measured head circumference values had an age range of 0–226 months (0–18.8 years).

To assess reliability, a test-retest dataset was used [31], consisting of 120 datasets from three healthy subjects (2 males), each of whom underwent 40 MRI exams over a period of 31 days [32]. Details on both datasets are provided in the supplementary material 1.

To further assess plausibility, the approaches’ different outcome measures (head and brain circumference) were generated from the validation dataset and related to each other, as a function of age.

**Fig 1:**
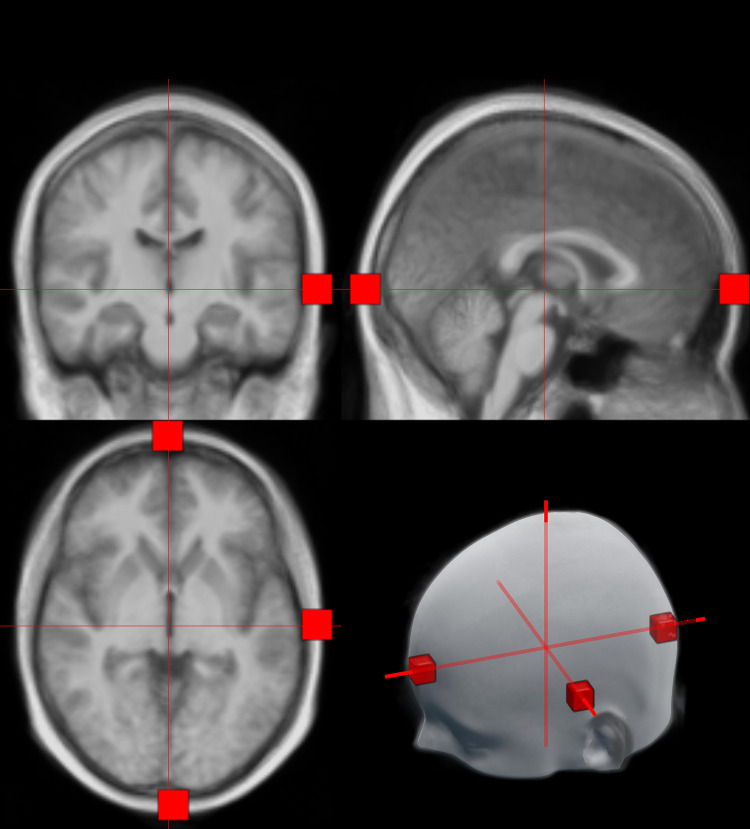
Reference brain & reference head with labelling of the regions of interest (ROIs). Location of the three ROIs in sagittal, coronal, and axial planes of the reference head: maximal frontal region (glabella), maximal occipital region (protuberantia occipitalis), and region above the left ear. Insert: surface rendering of the reference head: ROIs shown as red squares. Figure created with MRIcroGL [33].

For data processing, 3D T1 MRI datasets were used. The SPM12 software package (Wellcome Trust Centre for Neuroimaging, London, UK), running in MATLAB (Mathworks, Natick, MA, USA), was used to process the MRI datasets. Figures were created using MRIcroGL [33] (Fig 1) or using MATLAB functionality (all others).

### Definitions

Three anatomical landmarks were used to define occipitofrontal circumference: anteriorly, the maximum frontal area (glabella), supraorbital, at forehead level, and posteriorly, the maximum occipital protuberance [5,11,34]. These points were manually identified once on the reference brain used for spatial normalization (see “Processing”, below). A third point was placed at the same level as the frontal and occipital points, to the left above the ear, allowing to create a horizontal plane, as recommended [35]. Regions of interest (ROIs) were generated around each point with a diameter of ±5 voxels (i.e., 1 cm) in each dimension, as illustrated in Fig 1.

### Data preparation

Initially, each dataset was coregistered to the reference brain in order to optimize the starting conditions for subsequent data processing steps, using a rigid-body transformation [36] within the optimized approach by [37]. Following spatial normalization, images and segmentation results were visually inspected for quality control. To allow for comparison irrespective of spatial resolution of the input data, a voxel size of 1 × 1 × 1 mm was used.

### Data processing

“Unified segmentation” [38] was employed to achieve spatial normalization, segmentation, and bias correction. We employed a recently-developed approach to a more robust affine spatial normalization (which integrates seamlessly into the unified segmentation approach [39]); otherwise, default settings were used for warping, bias correction, spatial sampling and tissue priors. The spatial normalization parameters resulting from unified segmentation were then inverted and used to denormalize the standard-space ROIs created above (see Fig 1), thus defining the reference points in the individual native space. Using Otsu's thresholding method available within Matlab, the T1-weighted image was separated from the background, ensuring that only the head (and not artefacts) was used for head circumference measurements. The gray matter partition resulting from segmentation was written out in native space, allowing for the determination of brain circumference.

To compensate for head positioning differences, 40 tilts of the head around the x-axis were simulated. To achieve this, the head was rotated forward in a “nodding” fashion by 18 ° and was then iteratively rotated back to −18 ° in 40 steps. For each tilt step, the head was scanned axially from bottom to top, and a slice was considered when at least 2 out of three ROIs (anterior forehead, occiput and/or above the left ear) were present. Slices meeting this criterion were saved as “valid slices”.

Thereafter, automated head/brain circumference measurement was achieved using a convex hull approach implemented in Matlab’s bwconvhull function [40]. This generates an “imaginary loop” on axial slices along the outer head/brain circumference, imitating the behavior of a measuring tape. While this may, for example, include the ears, this corresponds to the use of the measuring tape in practice. Further, the initial outer line generated using bwconvhull was smoothed using a Savitzky-Golay filter [41]. Here, each data point is smoothed taking into account its surrounding points using a local polynomial [42]. The filter width describes the number of points to each side of the point to be smoothed that are described by the polynomial. Here, a polynomial degree of 2 was used, resulting in a smoother outline (since the original coordinates can only be determined exactly to the nearest full voxel position). The best filter width was determined by using no filtering and filter widths in steps of 10 from 10 to 120 (i.e., 13 filtering steps).

After performing these steps on the head, they were likewise repeated on the gray matter partition to obtain brain circumference. To save time and to ensure correspondence, brain circumference was only determined on slices previously found valid for head circumference.

The following analyses were performed: in a first step, the question of which summary indicator to use in the presence of several measurements (mean, median, or maximum, i.e., 3 indicators) was addressed, by analyzing the validation dataset using all filtering steps and all indicators. The results matrix (13 filtering steps by three indicators) was then summarized using the mean difference (bias) and the median absolute deviation (MAD) from the ground truth, and results were then compared statistically to find the best approach. The performance of this best approach in the validation dataset and as compared to the ground truth (manual measurement) was then visualized by using Bland-Altman analyses [43]. As suggested there, percentiles were used instead of standard deviations if the difference to the mean was found to be non-normally distributed.

As reference values, the (sex-averaged) 3^rd^ and 97^th^ percentile from a recent and large developmental dataset were used [44].

To assess the reliability of the approach, the test-retest-dataset (3 subjects with 40 MRI datasets each) was analyzed using the above-determined settings, and the stability of results across multiple measurements was assessed, again using Bland-Altman analyses.

To finally explore the biological plausibility of the approach, the automatically-derived head and brain circumferences from the validation dataset were compared with each other and as a function of age.

### Statistical analysis

All statistical analyses were carried out using MATLAB R2021a. An initial Kolmogorov-Smirnov-Lilliefors test was used to check for normal distribution. If the normal distribution was violated, a non-parametric Mann-Whitney U-test was used, otherwise a parametric T-test. All tests were two-sided. Significance was assumed at *p* ≤ 0.05. For multiple comparisons, Bonferroni correction was used to reduce the probability of type 1 errors.

To assess reliability in the test-retest dataset, Bland-Altman analyses were used again to illustrate agreement between measurements. As no gold standard was available here (head circumference was not known for the three subjects in the test-retest dataset), the mean of all measurements was instead used as the reference point per subject.

## Results

Comparison of different measurement variants: When comparing the performance of the three measurement variants (mean, median, or maximum value) across all 13 filtering steps in the validation dataset, the variant using the maximum value from all measurements performed best in both parameters (Table 1): the bias was significantly lower in the maximum variant than in the median variant (*p* = 0.0096; Mann-Whitney U-test, Bonferroni-corrected for two comparisons) and significantly lower in the maximum variant than in the mean variant (*p* = 0.00587; Mann-Whitney U-test, Bonferroni-corrected for two comparisons). Hence, the maximum variant was used in all further analyses.

**Table 1:** Performance of the three measurement variants (mean, median, maximum) across 13 filtering steps in the validation dataset (n = 153). Bias = mean difference, MAD = median absolute deviation. * = selected approach.

Approach	Bias [cm]	MAD [cm]
Mean	1.0678	1.3311
Median	1.0442	1.3226
Maximum *	0.5196	0.9847

Within the maximum variant approach, the best-performing filter setting was then identified. The best combination of both values (second-to-lowest bias and lowest median absolute deviation) was found for a Savitzky-Golay filter setting of 50, which was consequently used in all further analyses (Table 2).

**Table 2:** Effect of different Savitzky-Golay filter widths on the two performance measures in the validation dataset (n = 153), using the maximum approach. Bias = mean difference, MAD = median absolute deviation. * = selected approach.

Filtering width	Bias [cm]	MAD [cm]
**0**	0.7849	1.0370
**10**	0.9730	1.0427
**20**	1.0130	1.1144
**30**	0.8080	0.9487
**40**	0.3912	0.8393
50 *	0.0437	0.8349
**60**	−0.0863	0.8497
**70**	−0.0059	0.8791
**80**	0.2533	0.9477
**90**	0.5348	1.0281
**100**	0.6691	1.1051
**110**	0.6969	1.1021
**120**	0.6785	0.9968

**Fig 2:**
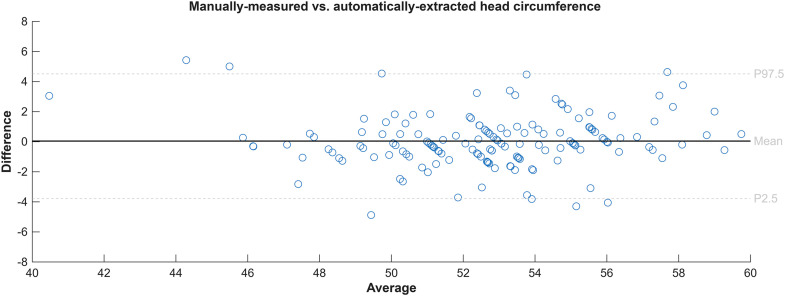
Bland-Altman plot comparing the manually measured head circumference with the automatically-extracted values. Confidence interval is designated by gray lines (percentiles 2.5 and 97.5).

With these settings, the mean difference (bias) in the Bland-Altman analysis was 0.044 cm (0.083%), with a median absolute deviation of 0.8349 cm. When visually assessing the Bland-Altman plot (Fig 2), no systematic bias could be observed.

**Fig 3:**
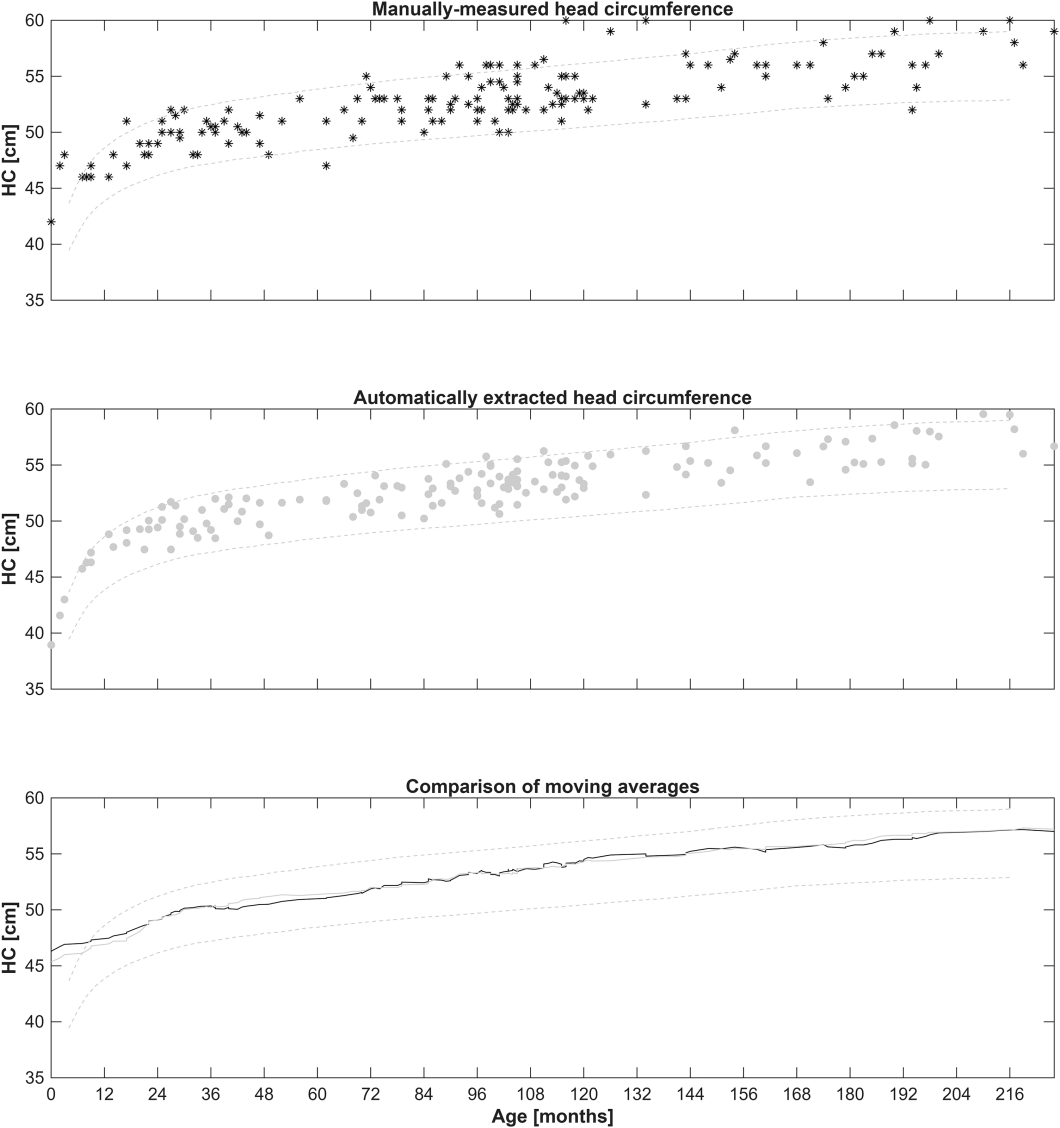
Gold standard (manually measured head circumference, top) versus MRI algorithm results (middle) as a function of age in the validation dataset (n = 153). The upper graphs show the individual data (black stars = manual measures, grey circles = MRI algorithm); in the lower graph, the respective trends are described by a moving average filter (black line = manual measures, grey line = MRI algorithm; filter width n = 20).

Gold standard versus MRI algorithm as a function of age: Results of the gold standard (manually measured head circumference) and our MRI algorithm are shown in Fig 3, as a function of age. For measures of agreement, see Tables 1 and 2 and Fig 2.

**Fig 4:**
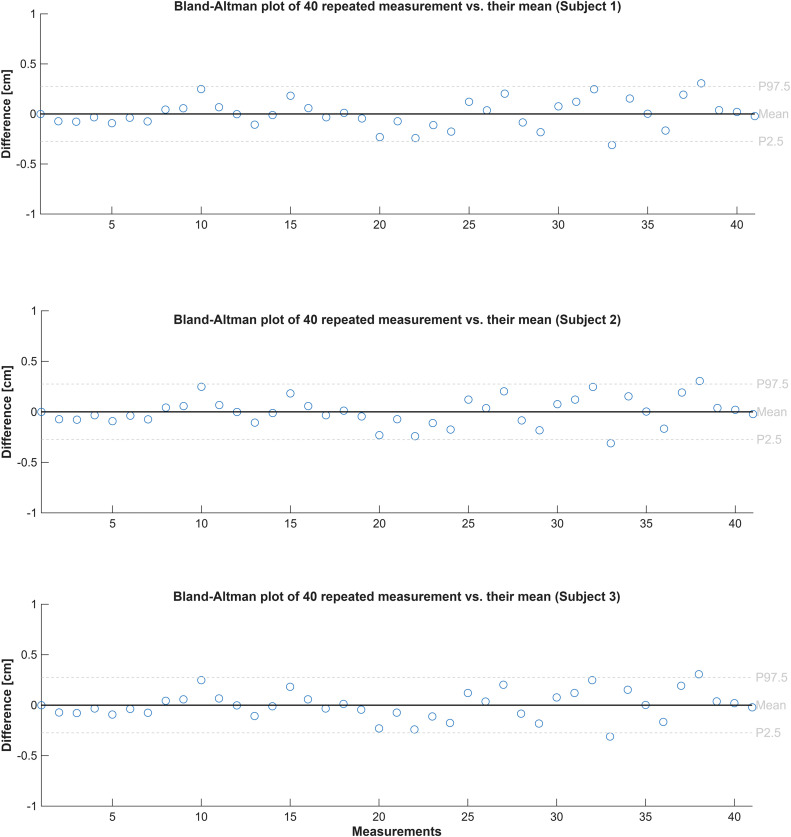
Test-retest dataset (40 MR datasets each from 3 subjects). Bland-Altman plots for each of the three subjects.

Reliability: The test-retest dataset was used to assess the reliability of the algorithm. Across all three subjects, the maximum range across all 40 measurements was 0.83 cm, with a standard deviation of 0.1–0.2 cm for all test datasets. Table 3 shows the descriptive statistics. In the Bland-Altman analysis of subject 1, comparing the 40 datapoints versus its mean, the median absolute deviation was 0.076 cm. For subject 2, MAD was 0.11 cm, and for subject 3, MAD was 0.095 cm. Fig 4 presents these results graphically, with again no evidence for a systematic bias.

**Table 3:** Descriptive statistics of the test-retest dataset.

	Subject 1 (n = 40)	Subject 2 (n = 40)	Subject 3 (n = 40)
Mean [cm]	59.13	58.13	55.62
Standard deviation [cm]	0.14	0.15	0.17
Median [cm]	59.14	58.11	55.59
Minimum [cm]	58.82	57.87	55.27
Maximum [cm]	59.44	58.47	56.11

**Fig 5:**
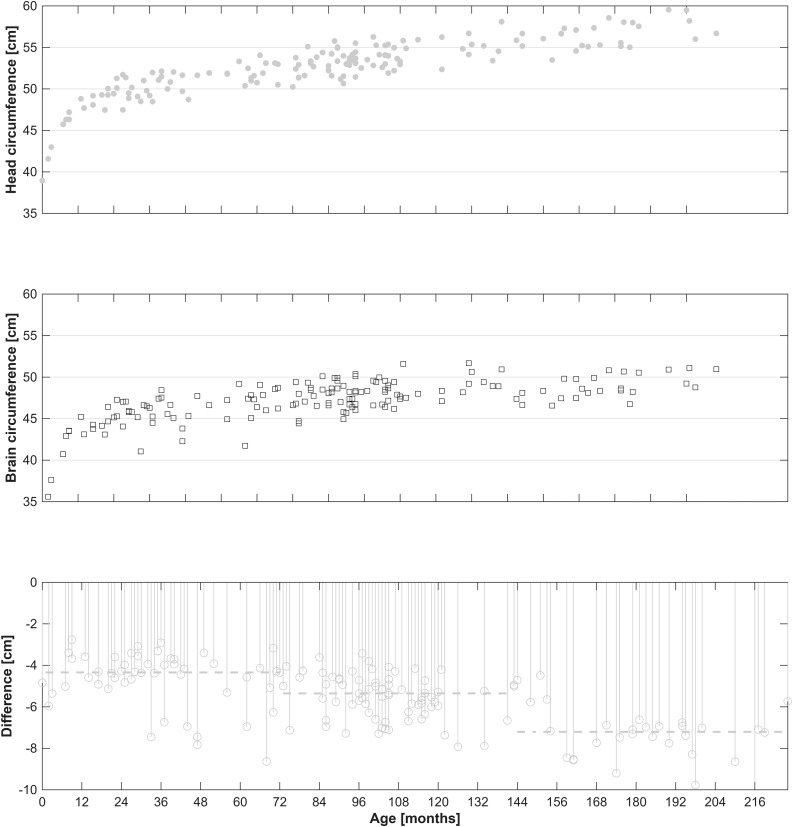
Comparison of head and brain circumference in the validation dataset (n = 153) as a function of age. Note similar trends in infancy but then further increases in head circumference while brain circumference plateaus, widening the gap between them. Upper plot shows head circumference (grey circles), middle plot shows brain circumference (black squares), bottom plot shows difference (in cm). Dotted lines in bottom plot are median values from 0-6, 6-12, and 12-18 years of age [44].

Plausibility: Head and brain circumference: The relationship between head and brain circumference as a function of age is shown in Fig 5. Here, phases with different growth rates are apparent: in the first months of life, there is a parallel steep increase in head and brain circumference. However, brain circumference values reach a plateau, while head circumference values continue to increase. There is a clear age dependency of this difference: when looking at age brackets, the median difference between head and brain circumference (dotted lines in bottom plot) was −4.34 [MAD 0.67] cm in children from 0‑6, ‑5.35 [MAD 0.68] cm in children 6–12, and −7.21 [MAD 0.54] cm in children 12–18 years of age. All these differences were significant (all *p* < 0.001; p-value Bonferroni-corrected for three comparisons, Mann-Whitney U-test).

## Discussion

This study investigated a new, automated approach to extract head circumference from MRI datasets of healthy subjects. Its validity (compared to the gold standard of manual measurement) and reliability (by analyzing repeated measurements) were examined. In addition, brain circumference was calculated as a new parameter and its biological plausibility was explored.

### Validity

Validity provides information on whether a measurement method – in this case the algorithm – measures what it is supposed to measure, and therefore provides credible results [45]. This question was investigated using a validation dataset for which both MRI data and manually measured head circumference values were available, from a total of 153 subjects aged 0–226 months (0–18.8 years). Our automatically-obtained head circumference values were compared with those of the gold standard.

Comparison of different summarizing indicators: in the literature, different approaches are suggested if more than one manual head circumference measurement is available, such as using the mean [4,13,46–49] or the maximum value [15–17]. Other authors found no significant differences between using “only one measurement,” “maximum value from two measurements” and “mean value from two or three measurements” [50].

In our data, we found that the variant using the maximum of several values performed significantly better than the other approaches (Table 1), which is consistent with recommendations for the procedure of manual measurement (e.g., [16]). For this reason, the maximum value across measurements was used in this work.

Performance of the algorithm with regard to the gold standard: The maximum value approach showed an extremely low bias (0.04 cm) with the best Savitzky-Golay-filter setting of 50 (Table 2), indicating that no systematic deviation is to be expected. The median absolute deviation was 0.83 cm. This is within the range of observed errors of measurements in the clinical field: for example, while an inter-observer difference of > 0.5 cm was considered unacceptable, it was present in up to 26% of measurements seen by [51]. A median absolute deviation between manual measurements of up to 0.78 cm was also seen by [52] (see also reliability, below). These results demonstrate that the validity of the algorithm is very similar to that of the gold standard.

Both parameters are also depicted as a function of age in Fig 3. Interestingly, when additionally plotting anthropometric reference values from [44], several discrepancies seem present. For example, several subjects show extremely high manually-measured values (e.g., at age 2 months, manual value 47 cm; at age 116 months, manual value 60 cm; at age 134 months, manual value 60 cm), each of which corresponds to a head circumference percentile of > 99 each [53,54]. The corresponding algorithm outputs were 41.57, 55.36 and 56.24 cm, each safely within the normal range. Also, the automatically-determined values for the very young babies (middle panel) correspond better with the expected values from the reference dataset (gray lines), which may also suggest uncertainties with manual head circumference measurement or documentation. Of note, when summarizing over 20 subjects (lower panel in Fig 3), the curves are virtually indistinguishable, underlining the minimal systematic bias and suggesting that the algorithm will be particularly useful for group studies.

### Reliability

These analyses were intended to test the reliability, i.e., the dependability or accuracy of measurement of the algorithm when used multiple times [55]. A dataset of three subjects with 40 MR examinations each was available for this purpose [31]. It is noteworthy that the algorithm was not repeatedly run on the same dataset, but run 40 times on 40 different datasets of the same subject, acquired over a period of several weeks. Hence, the variability due to different positioning in the scanner, differences in field homogeneity etc. is already inherent in our results.

The values obtained from these repeated analyses showed a very high degree of agreement. Bland-Altman analyses demonstrate that 95% of the measured values were within a corridor of 0.43 to 0.29 cm around the mean value; the corresponding standard deviation was 0.17 at most (cf. Table 3). In light of the known high inter-individual variance of manual measurements of head circumference, these are very low values. For example, an intra-examiner variability of at least 0.5 cm was seen in under 10% of cases and a corresponding interobserver variability in under 16% of cases [51]. Other authors observed a standard deviation of 0.6 cm in repeated measurements [47]. A similar standard deviation was also seen by others [52], despite the fact that the measurements were taken under optimal conditions and therefore likely underestimated real-world variability. Overall, therefore, these results demonstrate that the repeated application of the approach to repeated measures will yield very stable results.

### Plausibility: Head and brain circumference

The new option to determine not only head circumference, but corresponding brain circumference (i.e., the gray matter hull perimeter) from MR images is intriguing as the latter may help to differentiate abnormal head from abnormal brain growth. Our results clearly demonstrate that the correspondence between the two measures is high, but their relation changes as a function of age (Fig 5). In infancy, they are closer, while later-on, brain circumference plateaus while head circumference continues to increase. The absolute difference between them changes significantly as a function of age, from 4.34 cm in children < 6 to 7.21 cm in children > 12. Our algorithm can therefore be used to supplement the existing percentile curves for head circumference with percentile curves for brain circumference, and publicly available datasets with brain MR data (total n > 45.000) could be used to this effect [56–58]. This would be of particular clinical benefit in the case of unusual discrepancy, for example in the entity of benign enlargement of subarachnoid space in infancy, BESS [25,59]. Here, children show a temporary and benign increase in head circumference, usually in the first year of life, due to extracerebral (usually frontal) accumulation of CSF. These two defining aspects (temporary and benign), however, can only be determined in hindsight. In such cases in particular, a normal brain circumference despite a large head circumference could help to confirm the diagnosis and thus avoid unnecessary diagnostic or therapeutic steps. In how far the brain circumference parameter may be of use in other specific physiological or pathological scenarios will have to be the subject of future studies.

### Limitations

The approach presented here relies on landmark definitions defined in standard space and their transfer into native space by inverting the automated spatial normalization procedure. This approach allows for the investigation of larger groups (as necessary for the generation of robust reference data), but may fail in the case of severely abnormal head configuration, where manual identification of such landmarks may be more exact. To prevent that, we already used a more robust and less biased version to spatial normalization [39]. The ROI size was derived empirically while developing the algorithmic approach. The effect of this was not formally evaluated, but while a larger ROI size will automatically lead to more slices being considered as “potentially valid”, it will also reduce spatial specificity. The chosen ROI size was a compromise, designed to be inclusive enough to yield numerous intersecting slices but exclusive enough to still be spatially specific. Regarding reliability analyses, it must be acknowledged that repeated measures of only three subjects were available, potentially limiting generalizability. Further limitations include the nature of a retrospective study with regard to demographic data (manually measured head circumference). This type of study does not allow retrospective verification of clinical measurements and it cannot be ruled out that incorrect information is included in the analyses (as discussed above in the case of the probable outliers). The current manuscript was aimed to establish and evaluate the approach in the setting of healthy brain development, but its performance in specific pathological settings was not investigated here. While in general terms we would expect that lower data quality (low resolution clinical images, motion or implant artefacts etc.) has the potential to impact segmentation, the unified segmentation approach used here is rather robust. Further, higher-order cranial deformities may disrupt the assumption of convexity, which may impair the applicability of the convex hull approach used here. For most “unusual brains”, however, the approach should be sufficiently robust, and the visualizations included in the algorithm allow to assess data processing and analysis success. Finally, several settings (number of tilts, tilt steps, etc.) were decided on empirically, and the effect of changing either has not been investigated systematically. However, they have worked well in our extensive test runs and simulations and while specific scenarios may call for different settings, we hope that by transparently providing this information herein and in the toolbox, the user will be able to adapt these settings if need be.

### Extensions

Based on the available data in the literature [44], the percentiles of head circumference can also be determined automatically if subject age and gender are provided. This functionality has now been implemented; an example can be found in the supplementary material 2. Additionally, other segmentation & normalization approaches such as implemented in CAT12 [60] can also be implemented.

## Conclusion

A new algorithm for the automated analysis of head circumference was shown to have a high validity (with respect to the current gold standard) and a very high level of reliability (when performing repeated measurements). The particular strength of the method is that very large MRI datasets could be analyzed and used to generate new insights by supplying a basis for a robust “description of normal” of not only head, but also brain circumference development, which in turn may allow for a more sensitive detection of abnormal development.

## Supporting information

S1 File Details regarding the validation and test-retest datasets used in this manuscript. (DOCX)

S2 File Example presentation of results including head circumference percentiles. First row: image positioning at the first, median and last tilt step, each in sagittal section; second row: percentile results for a boy aged 149 months: Head circumference in the range of the 99th percentile, corresponding to z = 2.21, (shown are the percentiles 3, 10, 25 as well as 75, 90 and 97); Third row: representation of the measured circumferences of the T1 dataset (circles, across all 189 slices) as well as the identification of the 10 slices that intersect at least 2 ROIs (“valid slices”; columns), resulting in a head circumference of 58.18 cm. Fourth row: representation of the measured circumferences of gray matter (circles) over only the slices that have already been identified as valid slices (columns), resulting in a brain circumference of 52.07 cm. HC = head circumference; GM = grey matter. From [61].
(PDF)
