# Supplementary material for: An automated approach to extracting head and brain circumference from MRI datasets
Source: PLoS One. 2026 Jul 27;21(7):e0352445. doi: 10.1371/journal.pone.0352445 (PMC13405294; doi:10.1371/journal.pone.0352445)
Supplement: S1 File — (DOCX) [file pone.0352445.s001.docx]

**An automated approach to extracting head and brain circumference from MRI datasets**

Jasmin Klischat ^1,2^ & Marko Wilke ^1,2*^

*^1^* *Department of Neuropediatrics, General Pediatrics, Diabetology, Endocrinology, Social Paediatrics, University Children's Hospital, Tübingen, Germany*

*^2^ Experimental Pediatric Neuroimaging, Children’s Hospital and Department of Neuroradiology, University of Tübingen, Germany*

* Corresponding author

E-mail: Marko.Wilke@med.uni-tuebingen.de (MW)

**Supplementary material 1: detailed listing of subject IDs from all datasets**

**Dataset 1**: From the Cincinnati MR Imaging of NeuroDevelopment (C-MIND) study, URL: https://nda.nih.gov/edit_collection.html?id=2329, 153 subjects with the following IDs were used: 00F011_C, 00M002_U, 00M018_C, 00M065_C, 00F037_C, 00F020_C, 00M002_C, 00M034_C, 01M001_C, 01F032_C, 01F033_C, 01F015_C, 01F036_C, 01M002_U, 01M008_C, 02F003_C, 02F023_C, 02M005_U, 02F031_C, 02M034_C, 02F020_C, 02F029_C, 02M022_C, 02M015_C, 02F043_C, 02F028_C, 02F021_C, 02F044_C, 02F035_C, 02F016_C, 03M033_C, 02M021_C, 03M005_C, 03M017_C, 03F026_C, 03M034_C, 03F034_C, 03F003_C, 03F017_C, 04F022_C, 04F019_C, 04F001_C, 05F017_C, 05M004_C, 05F016_C, 05F015_C, 05M002_C, 05F006_C, 05M001_C, 05F012_C, 06F014_C, 06M018_C, 06M011_C, 06F016_C, 06M007_C, 06F015_C, 06M014_C, 06M008_C, 07F004_C, 07M005_C, 07F001_C, 07F012_C, 07M007_U, 07M008_U, 07M006_U, 07F002_U, 07M014_C, 07M009_C, 07F010_C, 07F002_C, 07M017_C, 08F020_C, 07M005_U, 07M008_C, 08F013_C, 08M008_C, 08M002_U, 08F010_U, 08F016_C, 08M011_C, 08F007_U, 08F005_U, 08F010_C, 08M002_C, 08M012_C, 08F005_C, 08M003_U, 09M005_C, 08F006_C, 08F008_C, 08F006_U, 08F009_U, 08F017_C, 08F021_C, 08M004_U, 08M017_C, 08F008_U, 08F002_U, 09F004_U, 09M003_U, 09M007_C, 09M004_C, 09F010_U, 09F006_U, 09M004_U, 09M014_C, 09M021_C, 09F017_C, 09M002_U, 09M005_U, 09F005_U, 09F009_C, 09F007_U, 09F009_U, 09M001_U, 09F012_C, 10F002_C, 10F006_C, 11F007_C, 11M005_C, 11F004_C, 11F008_C, 11M007_C, 12M002_C, 12F007_C, 12M007_C, 12F004_C, 12F005_C, 13F005_C, 13F003_C, 13F004_C, 14F006_C, 14F003_C, 14F002_C, 14M012_C, 14F009_C, 14M014_C, 15M022_C, 15M015_C, 15F001_C, 15F002_C, 15M013_C, 16F007_C, 15M005_C, 16M010_C, 16F005_C, 16M007_C, 16M014_C, 17M010_C, 17M003_C, 18M001_C, 18F004_C, 18F012_C.

Please note: data presented in this work was obtained from the database known as Cincinnati MR Imaging of NeuroDevelopment (C-MIND), provided by the Pediatric Functional Neuroimaging Research Network. This Network and the resulting C-MIND database was supported by contract from the Eunice Kennedy Shriver National Institute of Child Health and Human Development (HHSN275200900018C).

**Dataset 2:** From *Maclaren, Julian; Han, Zhaoying; B. Vos, Sjoerd; Fischbein, Nancy; Bammer, Roland; Feng, Xy (2014). Test-Retest Reliability of Brain Volume Measurements.* URL: https://doi.org/10.6084/m9.figshare.929651_D7, 40 MRI scans each of 3 subjects with the following IDs were used: Subject 1 (M/26y), Subject 2 (M/31y) and Subject 3 (F/30y).
