# Supplementary figures and images for: An automated approach to extracting head and brain circumference from MRI datasets
Source: PLoS One. 2026 Jul 27;21(7):e0352445. doi: 10.1371/journal.pone.0352445 (PMC13405294; doi:10.1371/journal.pone.0352445)

First, median, and last image position

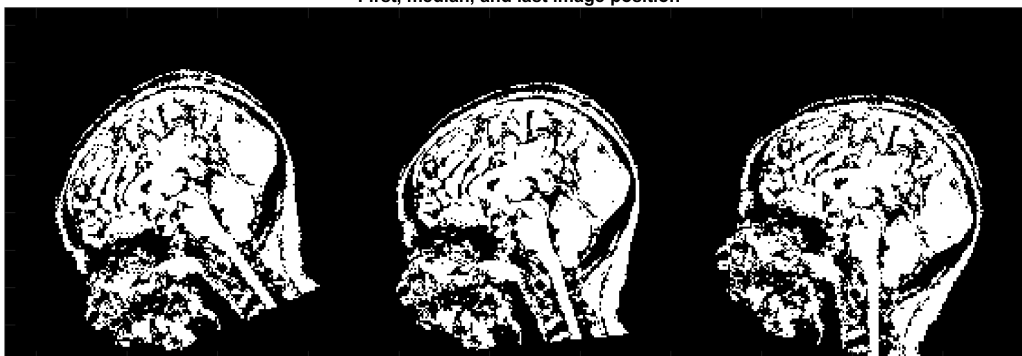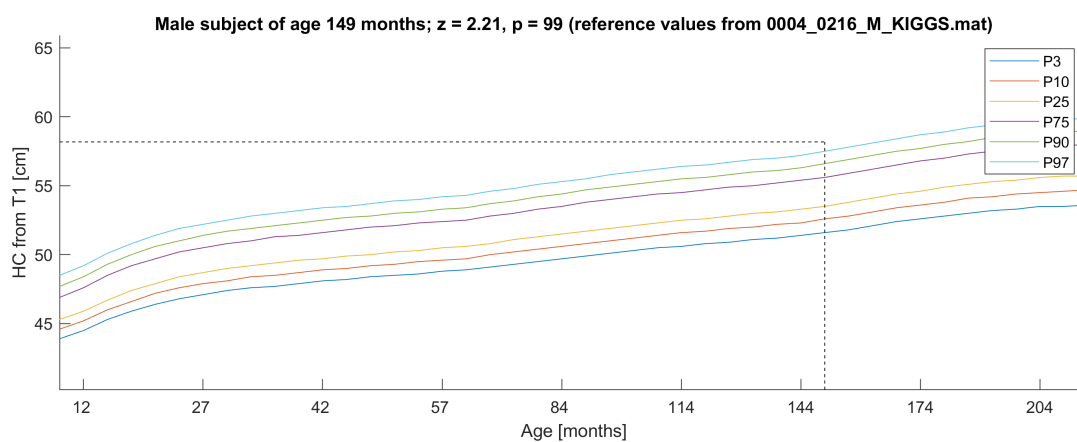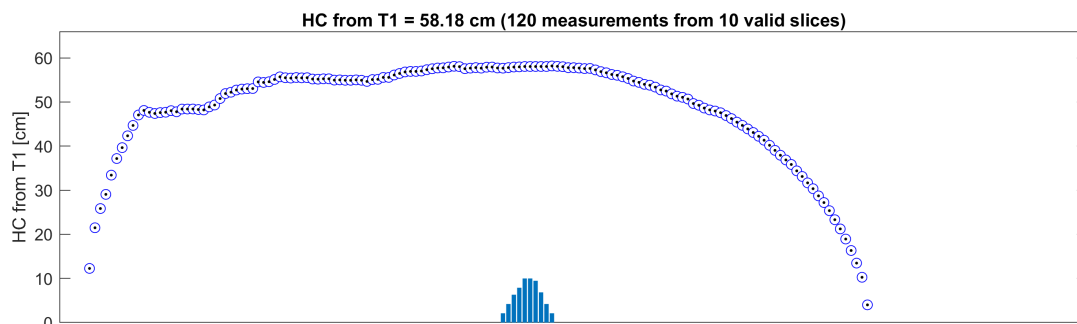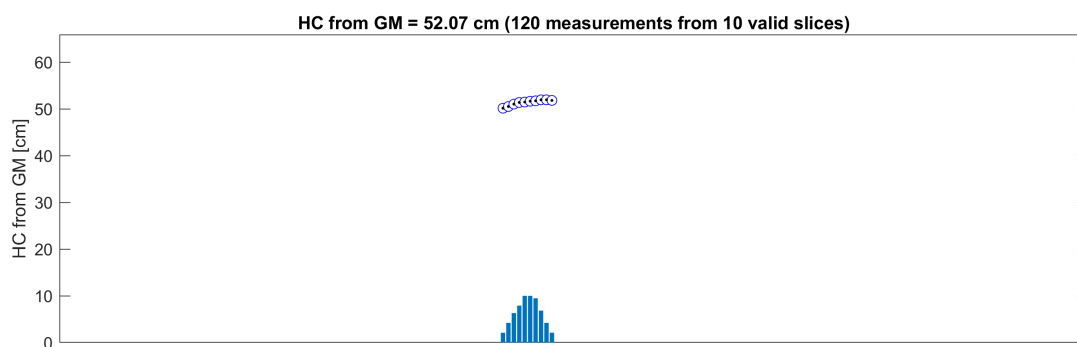

Axial dimension (slices 1:189, voxel size = 1.5 mm)

Supplement: S2 File — First row: image positioning at the first, median and last tilt step, each in sagittal section; second row: percentile results for a boy aged 149 months: Head circumference in the range of the 99th percentile, corresponding to z = 2.21, (shown are the percentiles 3, 10, 25 as well as 75, 90 and 97); Third row: representation of the measured circumferences of the T1 dataset (circles, across all 189 slices) as well as the identification of the 10 slices that intersect at least 2 ROIs (“valid slices”; columns), resulting in a head circumference of 58.18 cm. Fourth row: representation of the measured circumferences of gray matter (circles) over only the slices that have already been identified as valid slices (columns), resulting in a brain circumference of 52.07 cm. HC = head circumference; GM = grey matter. From [61]. (PDF) [file pone.0352445.s002.pdf]
